# A Novel Cyclic Pentadepsipeptide, *N*-Methylsansalvamide, Suppresses Angiogenic Responses and Exhibits Antitumor Efficacy against Bladder Cancer

**DOI:** 10.3390/cancers13020191

**Published:** 2021-01-07

**Authors:** Jun-Hui Song, Juhee Park, Sung Lyea Park, Byungdoo Hwang, Wun-Jae Kim, Chan Lee, Sung-Kwon Moon

**Affiliations:** 1 College of Biotechnology and Natural Resources, Chung-Ang University, Anseong 17546, Korea; goodabc123@cau.ac.kr (J.-H.S.); ajzzang99@naver.com (S.L.P.); byungdoo0409@naver.com (B.H.); 2 Advanced Food Safety Research Group, BrainKorea21 Plus, Department of Food Science and Technology, Chung-Ang University, Anseong 17546, Korea; bjhwngml@naver.com; 3 Department of Urology, Chungbuk National University, Cheongju, Chungbuk 28644, Korea; wjkim@chungbuk.ac.kr

**Keywords:** MSSV, bladder cancer, anti-tumor efficacy, anti-angiogenesis, single oral dose of acute toxicity

## Abstract

**Simple Summary:**

We found a novel cyclic pentadepsipeptide, N-methylsansalvamide (MSSV), and evaluated its anti-tumor action against bladder cancer using in vitro and in vivo model systems. Additionally, we report its anti-angiogenic responses both in vitro and in vivo. Moreover, acute toxicity test and tissue staining for liver function revealed that orally administered MSSV (2000 mg/kg for 14 days) exerted no harmful effects as it did not cause animal death, undesirable weigh alteration, adverse clinical symptoms, and abnormal biochemical marker levels (AST, ALT).

**Abstract:**

Here, we explored the anti-tumor efficacy of a cyclic pentadepsipeptide, *N*-methylsansalvamide (MSSV), in bladder cancer. MSSV inhibited the proliferation of both bladder cancer 5637 and T24 cells, which was attributed to the G1-phase cell cycle arrest, apoptosis induction, and alteration of mitogen-activated protein kinases (MAPKs) and protein kinase b (AKT) signaling pathways. Additionally, the treatment of bladder cancer cells with MSSV suppressed migratory and invasive potential via the transcription factor-mediated expression of matrix metalloproteinase 9 (MMP-9). MSSV abrogated vascular endothelial growth factor (VEGF)-induced angiogenic responses in vitro and in vivo. Furthermore, our result showed the potent anti-tumor efficacy of MSSV in a xenograft mouse model implanted with bladder cancer 5637 cells. Finally, acute toxicity test data obtained from blood biochemical test and liver staining indicated that the oral administration of MSSV at 2000 mg/kg caused no adverse cytotoxic effects. Our preclinical data described the potent anti-angiogenic and anti-tumor efficacy of MSSV and showed no signs of acute toxicity, thereby suggesting the putative potential of oral MSSV as a novel anti-tumor agent in bladder cancer treatment.

## 1. Background

Bladder cancer is considered a critical malignancy worldwide. Approximately, 90% of the bladder cancers manifest as transitional cell carcinoma (TCC), which are categorized as non-muscle invasive bladder cancer (NMIBC) and muscle invasive bladder cancer (MIBC) [1,2,3]. Although the common treatment, including transurethral resection, intravesical immunotherapy, and chemotherapy, is effective in the patients with NMIBC, depending on the tumor heterogeneity and clinical stage and grade, the tumors tend to recur and progress into an invasive disease such as MIBC [3]. Maximum mortality cases are encountered among patients with MIBC [3]. Therefore, the development of effective strong and safe novel drugs for MIBC treatment is urgently needed. 

The main process in the development and progression of bladder cancer is strongly associated with proliferation and metastasis of tumor cells [3]. Intensive research has demonstrated that cancer cells proliferated via regulation of control of cell cycle, apoptosis-related factors, and activation of MAPK and AKT signaling pathways [4,5,6]. Metastasis potential is responsible for the migration and invasion of tumor cells [3,7]. Angiogenesis is a primary prerequisite pathological phenomenon during proliferation and metastasis of tumor cells [8]. A number of anti-cancer drugs have been developed to estimate the tumor-suppressive efficacy of the target molecules [9]. However, a highly efficacious, safe, and novel agent for bladder tumor treatment has not yet been found.

Cyclic depsipeptides, one variety of secondary metabolites produced by fungi, bacteria, and plants, are known to possess numerous pharmacological properties, such as antifungal, antibacterial, immunomodulatory, and antitumor properties [10,11]. Sansalvamide analogs are the new cytotoxic cyclic pentadepsipeptide present in marine plants, namely *Fusarium* species [11]. Sansalvamide A, which is a cyclic pentadepsipeptide found in *Halodule wrightii,* exhibited a molecular structure containing one hydroxy acid (leucic acid (*O-*Leu)) and four hydrophobic amino acids (two leucines (Leu), phenylalanine (Phe), and valine (Val)) [11,12]. Additionally, *N*-methylsansalvamide, which was purified from green algae in ocean, had a molecular structure comprising one hydroxy acid (leucic acid (*O-*Leu)) and four amino acids (phenylalanine (Phe), leucine (Leu), *N-*methyl-leucine (*N*-MeLeu), and valine (Val)) [11,13]. Both sansalvamide A and *N*-methylsansalvamide have reported a weak anticancer effect [11,12,13].

We identified a cyclic pentadepsipeptide, *N*-methylsansalvamide (MSSV), which was produced by *Fusarium* spp. isolated from Korean potato. MSSV (-*O*Leu-Val-*N*MeLeu-Phe-Leu) has a cyclic peptide structure containing a sansalvamide attached to four amino acids and one hydroxy acid. It has a similar molecular structure compared to that of a peptide previously isolated from cultures of a marine Fusarium sp. found in green algae [11,13]. A previous study revealed in vitro cytotoxic effects of cyclic depsipeptides including sansalvamide, *N*-methylsansalvamide, and their analogue [10,11,12,13,14]. However, the molecular effect and safety of MSSV obtained from land plant-associated fungus as a strong chemotherapeutic agent against cancer has not yet been investigated. Here, to our knowledge, the anti-tumor efficacy, anti-angiogenic effect, and single-dose acute toxicity of MSSV in cancer cells both in vitro and in vivo are reported for the first time.

## 2. Materials and Methods

### 2.1. Materials

Polyclonal antibodies specific to extracellular signal regulated kinase (ERK), phospho-ERK, p38MAPK, phospho-p38MAPK, c-Jun N-terminal kinase (JNK), phospho-JNK, AKT, and phospho-AKT were obtained from Cell Signaling (Danvers, MA, USA). U0126, SP600125, SB203580, and LY294002 were obtained from Calbiochem (San Diego, CA, USA). Polyclonal antibodies against cyclin D1, cyclin E, CDK2, CDK4, p53, p21WAF1, p27KIP1, and GAPDH were obtained from Santa Cruz Biotechnology Inc. (Santa Cruz, CA, USA). Polyclonal antibodies specific to FAS, B-cell lymphoma 2 (Bcl-2), Bcl-2-associated X protein (Bax), X-linked inhibitor of apoptosis protein (XIAP), poly(ADP-ribose) polymerase-1 (PARP-1), caspase-3, caspase-6, caspase-7, caspase-8, caspase-9, and actin were purchased from Santa Cruz Biotechnology (Santa Cruz, CA, USA) and Cell Signaling (Danvers, MA, USA). Polyclonal antibodies to phospho-endothelial nitric oxide synthase (eNOS) (S1177) and eNOS were obtained from Cell Signaling (Danvers, MA, USA). Human recombinant VEGF was purchased from Research and Diagnostic Systems, Inc. (Minneapolis, MN, USA).

### 2.2. Production of MSSV and Structure Elucidation

MSSV was obtained similarly as described in the previous studies [10,12]. Briefly, MSSV was produced by cultivation of *Fusarium* spp. KCCM12601P isolated from Korean potato in the *Fusarium* defined media agar at 25 °C for 10 days. The culture was extracted with same volume of chloroform by agitation at 150 rpm for 6 h (25 °C). The extracted solvent was filtrated through Whatman No. 4 filter and the filtrate was evaporated to dryness at 36 °C using rotary evaporator. The residue was dissolved in HPLC-grade methanol and the solution was filtered through a Whatman PVDF filter (pore size 0.45 μm). MSSV was further purified by recycling preparative HPLC (Japan Analytical Industry Co. Ltd., Tokyo, Japan) equipped with UV detector (Japan Analytical Industry Co. Ltd., Tokyo, Japan) and fraction collector (Japan Analytical Industry Co. Ltd., Tokyo, Japan) using PrepHT XDB-C18 column (21.2 × 250 mm, 7-micron, Agilent, Pal Alto, CA, USA) at 25 °C. A mixture of acetonitrile and water (90:10, *v*/*v*) was applied as mobile phase (flow rate of 6 mL/min) and MSSV peak was monitored at 210 nm. MSSV powder was obtained from the collected solution after concentration with rotary evaporator (Eyela, Tokyo Rikakikai Co. Ltd., Tokyo, Japan).

The molecular formula of MSSV was assigned as C_33_H_52_N_4_O_6_ (obsd (M + H)^+^ as *m*/*z* 600.43) by high-resolution electrospray ionization mass spectra (HR-ESI-MS) and by using Waters Synapt G2 mass spectrometer (Waters, city, Milford, MA, USA). ^1^H-(400 MHz), ^13^C-(100 MHz) NMR spectra were recorded on a Bruker Avance II 400 MHz NMR spectrometer (Karlsruhe, Germany) in ppm relative to that of tetramethylsilane (TMS) as an internal standard (*J* in Hz) at 294 K.

### 2.3. Cell Culture

The human bladder carcinoma cell lines, 5637 and T24, were purchased from the American Type Culture Collection (Manassas, VA, USA) and maintained in Dulbecco’s modified Eagle medium supplemented with 10% fetal bovine serum (FBS), 100 U/mL penicillin, and 100 μg/mL streptomycin at 37 °C in a 5% CO_2_ humidified incubator. The 5637 cells were referred to as MGH-U1 cells. Additionally, primary human umbilical vein endothelial cells (HUVECs) were obtained from Lonza (Walkersville, MD, USA). Cells were grown on plates coated with 0.1% gelatin (Sigma, San Diego, CA, USA) in endothelial basic medium (EBM) and cultured in endothelial growth medium-2 (EGM^TM^ 2) Bulletkit^TM^ (Lonza) at 37 °C in a 5% CO_2_ humidified incubator. All experiments were performed between passages 2 and 5.

### 2.4. Cell Viability

Cell viability was performed using a modification of the 3-(4,5-dimethylthiazol-2-yl)-2,5-diphenyltetrazolium bromide (MTT) assay. Briefly, cells were plated in 96-well plates (6 × 10^3^ cells/well), followed by incubation with MSSV (0, 10, 20, and 30 µg/mL). After 24 h incubation, the plate was washed and fresh medium containing 10 μL of 5 mg/mL MTT was added. After 1 h, the medium was removed and replaced with 100 μL of dimethyl sulfoxide (DMSO). Absorbance at 540 nm was measured using a fluorescent plate reader.

### 2.5. Cell Counting

Cells were plated in 6-well plates and treated with MSSV (0, 10, 20, and 30 µg/mL) for 24 h. The cells were removed from the plates by treatment with 0.25% trypsin containing 0.2% EDTA (Corning, NY, USA). Separated cells were mixed with 50 μL of 0.4% trypan blue (Sigma-Aldrich, St. Louis, MI, USA) by gentle pipetting. Then, 20 μL of the mixture was loaded into each chamber of a hemocytometer and counted.

### 2.6. Cell Cycle Analysis

Cells were harvested and fixed in 70% ethanol. After washing once with ice-cold phosphate buffered saline (PBS), cells were incubated with RNase (1 mg/mL) followed by propidium iodide (50 mg/mL). The phase distribution of the cell cycle was analyzed using a flow cytometer (FACStar, BD Biosciences, San Jose, CA, USA) equipped with the BD Cell Fit software.

### 2.7. Apoptosis Assay

For the apoptosis assay, Cell Death Detection ELISA Plus Kit (Roche Diagnostics, Pleasanton, CA, USA) was used by measurement of histone-complexed DNA fragments. Briefly, cells were cultured and treated with various concentrations of MSSV in 96-well plates. After 24 h, collected cells were treated with lysis buffer and centrifuged at 12,000 rpm. Supernatants were transferred to a streptavidin-coated 96 well microplate and incubated with anti-histone antibody (biotin-labeled) and anti-DNA antibody (peroxidase-conjugated) for 2 h at room temperature. After washing with the plates, the absorbance was determined using precision microplate reader at 405 nm.

### 2.8. Immunoblotting and Immunoprecipitation

Cells were washed twice with cold PBS and freeze–thawed in 200 μL of lysis buffer (containing HEPES (pH 7.5), 50; NaCl, 150; EDTA, 1; DTT, 1; EGTA, 2.5; β-glycerophosphate, 10; Na_3_VO_4_, 0.1; NaF, 1; and PMSF, 0.1 (all in mmol/L); 10% glycerol; 0.1% Tween-20; 10 μg/mL leupeptin; and 2 μg/mL aprotinin). After the cells were scraped into 1.5-mL tubes, the lysates were incubated on ice for 10 min. The cells were then centrifuged at 10,000× *g* for 10 min at 4 °C. The amount of protein was determined using a BCA protein assay reagent kit (Thermo Fisher Scientific Inc., Waltham, MA, USA). An equal amount of protein (25 μg each) was loaded onto a sodium dodecyl sulfate (SDS, 0.1%)–polyacrylamide gel (10%) and resolved by SDS-polyacrylamide gel electrophoresis (SDS-PAGE) under denaturing conditions. The proteins were transferred onto nitrocellulose membranes (Hybond, GE Healthcare Bio-Sciences, Marlborough, MA, USA). After blocking with 5% skim milk, the membranes were incubated with primary antibodies for 12 h, followed by incubation with peroxidase-conjugated secondary antibodies for 90 min. The immunocomplexes were then detected using a chemiluminescence reagent kit (GE Healthcare Bio-Sciences, Marlborough, MA, USA). For immunoprecipitation analysis, equal amounts of cell lysates were incubated with the indicated antibodies at 4 °C overnight. Protein A-Sepharose beads (Santa Cruz Biotechnology Inc., Santa Cruz, CA, USA) were then added to the immunocomplexes, followed by incubation at 4 °C for 2 h. The immunoprecipitated complexes were washed with 1× lysis buffer three times, resuspended in SDS-PAGE sample buffer containing β-mercaptoethanol (Bio-Rad, Richmond, CA, USA), and separated by electrophoresis.

### 2.9. Wound Healing Migration Assay

Cells (3 × 10^5^/well) were plated in 6-well plates. Cells were pretreated with mitomycin C (5 μg/mL, Sigma #M4287) for 2 h to inhibit cell proliferation. The cell surface area was then scratched with a 2 mm-wide pipette tip. After washing with PBS three times, the plate was incubated with culture media in the presence or absence of MSSV (0, 10, 20, and 30 µg/mL) for 24 h. The recovery capacity of the MSSV-treated cancer cells migrating into the scratched area was measured and compared with that of the control cells. Cellular images were photographed under an inverted microscope at 40× magnification.

### 2.10. Boyden Chamber Invasion Assay

Invasiveness was estimated using an invasion assay kit (Cell Biolabs, San Diego, CA, USA), according to the manufacturer’s instructions. Briefly, 2.5 × 10^4^ cells were resuspended in serum-free culture medium and incubated with mitomycin C (5 μg/mL) for 2 h before being seeded in the upper chamber. The medium containing 10% FBS or VEGF was added to the lower chamber as a chemo-attractant. After 24 h, cells in the lower chamber were fixed, stained with 0.01% crystal violet in 20% ethanol, and photographed.

### 2.11. Zymography

The conditioned medium was obtained and electrophoresed on a polyacrylamide gel containing 0.25% gelatin. The gel was washed twice for 15 min at room temperature with 2.5% Triton X-100. Subsequently, the gel was incubated at 37 °C overnight in a buffer containing 150 mM NaCl, 50 mM Tris-HCl, and 10 mM CaCl_2_ and having a pH of 7.5. The gel was stained with 0.2% Coomassie blue and photographed on a light box. Proteolysis was detected as a white zone on a blue field.

### 2.12. Electrophoretic Mobility Shift Assay (EMSA)

Nuclear extracts were prepared with a Nuclear Extraction kit (Panomics, Fremont, CA, USA). Briefly, cells were harvested by centrifugation, washed, and resuspended in a buffer containing 10 mM HEPES (pH 7.9), 10 mM KCl, 1 mM DTT, 0.5 mM PMSF, 0.1 mM EDTA, and 0.1 mM EGTA. After incubation on ice for 15 min, the cells were mixed vigorously with 0.5% NP-40. The nuclear pellet was separated by centrifugation, followed by extraction in a buffer containing 20 mM HEPES (pH 7.9), 400 mM NaCl, 1 mM DTT, 1 mM PMSF, 1 mM EDTA, and 1 mM EGTA at 4 °C for 15 min. The nuclear extract (10–20 μg) was preincubated at 4 °C for 30 min with a 100-fold excess of an unlabeled oligonucleotide spanning the −79 position of the MMP-9 cis-acting element of interest. The sequences were as follows: AP-1, CTGACCCCTGAGTCAGCACTT; NF-κB, CAGTGGAATTCCCCAGCC; and Sp-1, GCCCATTCCTTCCGCCCCCAGATGAAGCAG. The reaction mixture was then incubated at 4 °C for 20 min in a buffer (25 mM HEPES buffer (pH 7.9), 0.5 mM EDTA, 0.5 mM DTT, 50 mM NaCl, and 2.5% glycerol) with 2 μg of poly dI/dC and 5 fmol (2 × 10^4^ cpm) of a Klenow end-labeled (^32^P ATP) 30-mer oligonucleotide, which spanned the DNA-binding site of the MMP-9 promoter. The reaction mixture was resolved by electrophoresis at 4 °C using a 6% polyacrylamide gel. The gel was exposed on to an X-ray film overnight. The gray values of the blots were measured using the Image-Pro Plus 6.0 software (Media Cybernetics, Rockville, MD, USA).

### 2.13. Colony Tube Formation Assay (HUVECs)

Colony tube formation by HUVEC on a Matrigel was performed as described previously [15]. Briefly, HUVECs treated with MSSV (0, 1, 2.5 and 5 µg/mL) were incubated in a BD Matrigel matrix growth factor reduced-coated 24-well plate. After 8 h of incubation, tube formation was observed using an inverted microscope (40× magnification) and quantified by measuring the length of tubes using Image-Pro Plus software.

### 2.14. Aortic Ring Assay

The mouse aortic ring angiogenesis assay was performed as previously described [15]. Briefly, the aortas isolated from C57BL/6 mice were cut into sections of 1–1.5 mm long rings, and individually embedded in the Matrigel pre-coated wells. The aortic rings were incubated with the growth medium containing VEGF (50 ng/mL) and MSSV (25 and 50 µg/mL) for 9 days. The sprouting of endothelial tubes was quantified using Image-Pro Plus software (Media Cybernetics, Rockville, MD, USA). All animal experiments were performed with the approval of the Animal Care and Use Committee of Chungbuk National University.

### 2.15. Plug In Vivo Assay

Matrigel plug angiogenesis assay was performed as described previously [15]. Briefly, a mixture of BD Matrigel matrix (0.5 mL) and heparin (50 unit/mL; BD Bioscience) was subcutaneously injected into C57BL/6 mice (aged 6–7 weeks) with VEGF (50 ng/mL) and MSSV (125 and 250 µg/mL). After 7 days, mice were euthanized and the Matrigel plugs were removed. Vascularization was determined by measuring the hemoglobin content of the Matrigel plugs using the Drabkin method (Drabkin reagent kit 525, Sigma-Aldrich, Louis, MO, USA) as well as the infiltrated endothelial cell count in the Matrigel plugs via CD31 antibody staining. All animal experiments were performed with the approval of the Animal Care and Use Committee of Chungbuk National University.

### 2.16. Immunochemistry (CD31)

Paraffin sections (5 µm thick) were deparaffinized and rehydrated. Nonspecific sites were blocked with a solution containing 5% bovine serum albumin in PBS (pH 7.4) for 1 h. The sections were incubated overnight at 4 °C with QD565 nanoparticle-conjugated CD31 antibody (1:100) and further incubated with a fluorescent secondary IgG antibody (1:300). Images were obtained using confocal laser scanning microscopy (Carl Zeiss LSM 510, Carl Zeiss, Jena, Germany).

### 2.17. Hematoxylin and Eosin (H&E) Staining

The transplanted tumor tissues from mice were removed, fixed with formalin, and embedded in paraffin. Paraffin sections (5 µm thick) were stained with hematoxylin and eosin. Sections were deparaffinized in xylene and rehydrated in a graded series of alcohol. The tissue sections were stained for 7 min in 10% hematoxylin (Sigma-Aldrich) and the cytoplasm was subsequently stained for 5 min in 1% eosin (Sigma-Aldrich). The sections were imaged using an inverted microscope (Leica, Wetzlar, Germany).

### 2.18. Xenograft Experiment

For the nude mice xenograft tumor assay, 3.6 ×10^7^ T24 cells/mL were suspended in 100 µL PBS and mixed with 100 µL BD Matrigel matrix (BD Biosciences, NJ, USA); this solution was then injected subcutaneously into the back of male BALB/c nude mice (7-week-old; weight, 23–28 g). The mice were randomly divided into five groups (*n* = 6) when the tumor volume reached an average size of 200–400 mm^3^. Tumor volume was measured every 2 or 3 days using Vernier calipers and calculated using the formula (Tumor volume (TV) = (W^2^ × L)/2). Vehicle alone (methanol), MSSV (1, 5, and 15 mg/kg) were orally administered daily for 15 days and cisplatin (5 mg/kg) was intraperitoneally injected daily for 12 days. The general condition and body weight of mice were monitored daily. Tumor inhibition rate (%) was calculated using the formula (mean tumor weight of vehicle group−mean tumor weight of experiment group)/mean tumor weight of vehicle group ×100).

### 2.19. Oral Acute Toxicity and Biochemical Marker (ALT, AST) Test

Acute oral toxicity study was conducted at the Biotoxtech Co., Ltd. (Ochang, South Korea) which follows the Regulation of Good Laboratory Practice (GLP), as inspected by the Ministry of Food and Drug Safety. This study was approved by the Institutional Animal Care and Use Committees of Biotoxtech Co., Ltd. (Approval No. 150048). In this study, the animals used were 6 week old specific pathogen free (SPF) CrljOri:CD1 (ICR) male (25.3–29.0 g, 10 animals) and female (21.6–25.0 g, 10 animals) mice. There were four groups of ICR mice (5 mice/sex/group). Vehicle (DMSO, 4 mL/kg) or MSSV (2000 mg/4 mL/kg) was administrated only once on day 0 and observed until 14 days after treatment. On day 0, general symptoms (types of toxic indications, times of toxic expression and recovery times) and any deaths were observed at 30 min and at 1, 2, 4, and 6 h after administration of MSSV. General symptoms were continuously recorded once daily until day 14. Body weight were measured on the day 0, 1, 3, 7, and 14 after administration. On the day 14, all mice were euthanized under isoflurane anesthesia, and blood was collected from the abdominal aorta. The weights were statistically analyzed by using SAS (version 9.3, SAS Institute Inc., USA). The level of aspartate transaminase (AST) and alanine aminotransferase (ALT) in serum samples were determined by the manufacturer’s instructions. H&E staining was performed on tissue samples.

### 2.20. Statistics

All experiments were independently performed at least three times. One-way ANOVA and Student’s *t*-test were used to analyze the statistical significance among groups. Differences were considered significant when *p* value was < 0.05.

## 3. Results

### 3.1. Identification of MSSV Produced by FUSARIUM spp. KCCM12601P

**Figure 1:**
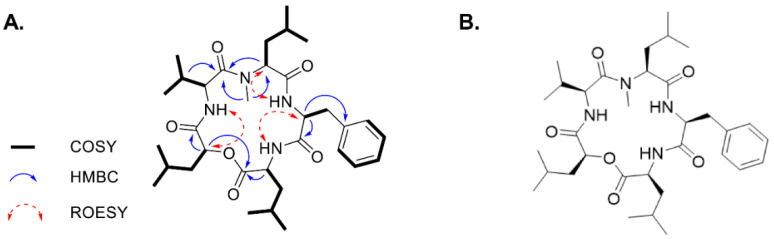
Structure elucidation of *N*-methylsansalvamide (MSSV). (**A**) Key correlation spectroscopy (COSY), HMBC, and ROESY correlations of MSSV. (**B**) Chemical structure of MSSV.

The molecular formula of MSSV was assigned as C_33_H_52_N_4_O_6_ (obsd (M + H)^+^ as *m*/*z* 600.43) by high-resolution electrospray ionization mass spectra (HR-ESI-MS). The chemical structure was elucidated by a combination of spectroscopic analysis based on the spectra of ^1^H-(400 MHz) and ^13^C-(100 MHz) NMR, including 1D, 2D NMR, and HR-ESI-MS. The detailed NMR data was described in Table S1. The connectivity of amino acids and a 2-hydroxy-4-methylpentanoic acid were identified by heteronuclear multiple-bond correlation spectroscopy HMBC, and rotating-frame nuclear Overhauser effect spectroscopy (ROESY) correlations (Figure 1A). These spectroscopic data led to the construction of the planar structure of the cyclic peptide as a reported cyclic depsipeptide MSSV (Figure 1B) [13].

### 3.2. MSSV Inhibits the Proliferation of Bladder Cancer Cells

**Figure 2:**
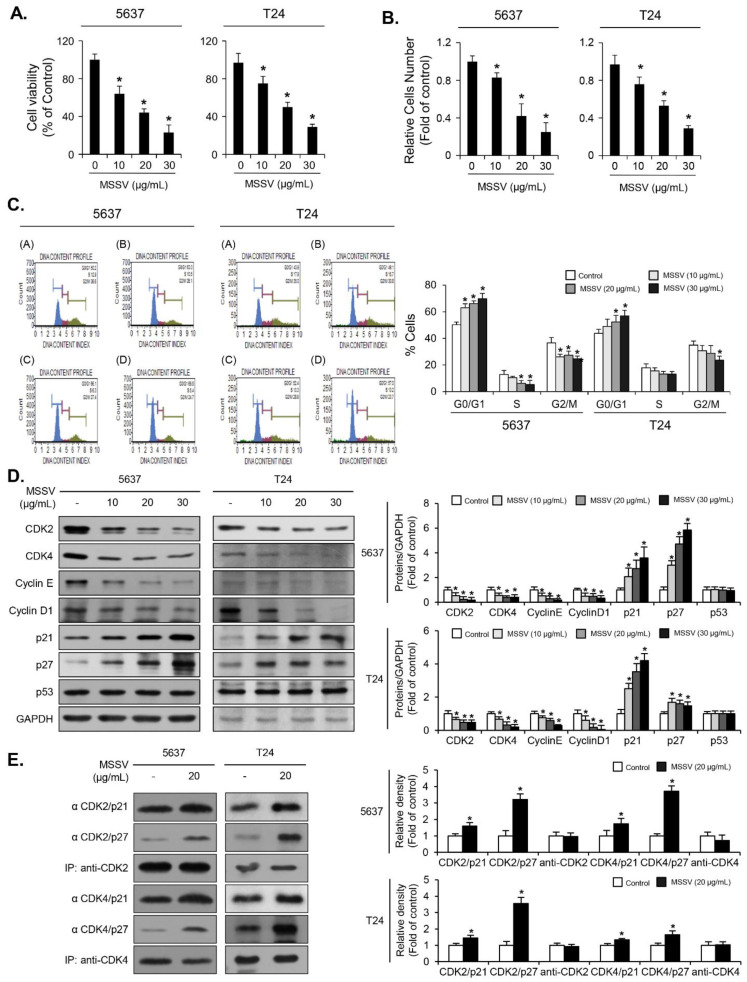
MSSV-mediated inhibition of proliferation of the human bladder cancer cell lines, 5637 and T24, was owing to G1-phase cell cycle arrest. (**A**) Both cell lines were treated with or without MSSV at the indicated concentrations for 24 h, followed by MTT assays for cell viability. (**B**) Cells were treated with indicated concentrations of MSSV for 24 h, and cell counting was performed via trypan blue staining. (**C**) Both cell lines were treated with MSSV for 24 h and analyzed for cell cycle distribution using fluorescence-activated cell sorting (FACS) histograms. The percentage of cells in each cell cycle phase is presented. (**D**) Cells were exposed to MSSV at the indicated concentrations for 24 h. The expression levels of cyclin D1, cyclin E, CDK2, CDK4, p21WAF1, p27KIP1, p53, and GAPDH were analyzed via immunoblotting. Bar graphs show the relative fold changes in proteins at different MSSV concentrations in comparison with the control. (**E**) The cell lysates were immunoprecipitated with antibodies recognizing CDK2 and CDK4, followed by immunoblotting with specific antibodies against p21WAF1, p27KIP1, CDK2, and CDK4. Graphs show the relative amount of immunoprecipitated proteins as fold changes in comparison with the control. For the bar graphs, values were presented as the mean ± SD of three independent experiments; * *p* < 0.05, compared with the control group. Uncropped Western Blot Images in Figures S3 and S4.

To investigate the anti-proliferative effect of MSSV, we performed a cell counting assay for invasive bladder cancer 5637 and T24 cells. Cells were treated with MSSV (10, 20, and 30 μg/mL) for 24 h. The proliferative ability of both cells decreased in a concentration-dependent manner (Figure 2B). Cell viability was determined by performing MTT assay. The viability of 5637 cells treated with MSSV reduced by 22%, 52%, and 71% at concentrations of 10, 20, and 30 μg/mL, respectively (Figure 2A), and the viability of MSSV-treated T24 cells reduced by 21%, 50%, and 72% at concentrations of 10, 20, and 30 μg/mL, respectively (Figure 2A). Concentrations of 10, 20, and 30 μg/mL were used for subsequent in vitro assays because IC50 values for both 5637 and T24 cells were 20 μg/mL.

### 3.3. MSSV Induces G1-Phase Cell Cycle Arrest of Bladder Cancer Cells

To understand the mechanism underlying cell proliferation inhibition induced by MSSV, we investigated its effects on 5637 and T24 cell cycle progression. As shown in Figure 2C, MSSV induced strong G1-phase cell cycle arrest in 5637 cells for 24 h as opposed to the vehicle. This G1-phase cell cycle arrest brought about by MSSV was accompanied with simultaneous reduction in cells in the S- and G2/M-phases (Figure 2C). Similar cell cycle arrest in the G1-phase owing to MSSV treatment was observed in T24 cells (Figure 2C). The percentage of cell population in each phase of the cell cycle after treatment with MSSV for 24 h is demonstrated in Figure 2C. These results indicate that MSSV caused a strong G1-phase arrest in bladder cancer cells.

### 3.4. MSSV Induces G1-Phase Cell Cycle Arrest via Decreased Expression of Cyclin/CDK and CDKI Induction in Bladder Cancer Cells

Because MSSV treatment blocked cell progression from the G1- to S-phase, we examined whether MSSV affects the expression of G1-phase cell cycle regulatory proteins. In both 5637 and T24 cells, MSSV treatment decreased the expression of cyclin D1, cyclin E, CDK2, and CDK4, but increased the expression of p21WAF1 and p27KIP1 (Figure 2D). Additionally, p21WAF1 level in CDK2 and CDK4 complexes in MSSV-treated bladder cancer cells was higher than that in the untreated cells (Figure 2E). p27KIP1 level associated with CDK2 and CDK4 also increased in both MSSV-treated bladder cancer cells as opposed to the untreated cells (Figure 2E). These results demonstrated that both p21WAF1 and p27KIP1 contribute toward G1-phase cell cycle arrest by binding to CDK2 and CDK4 in MSSV-treated bladder cancer cells.

### 3.5. MSSV Induces MAPKs and AKT Phosphorylation in Bladder Cancer Cells

**Figure 3:**
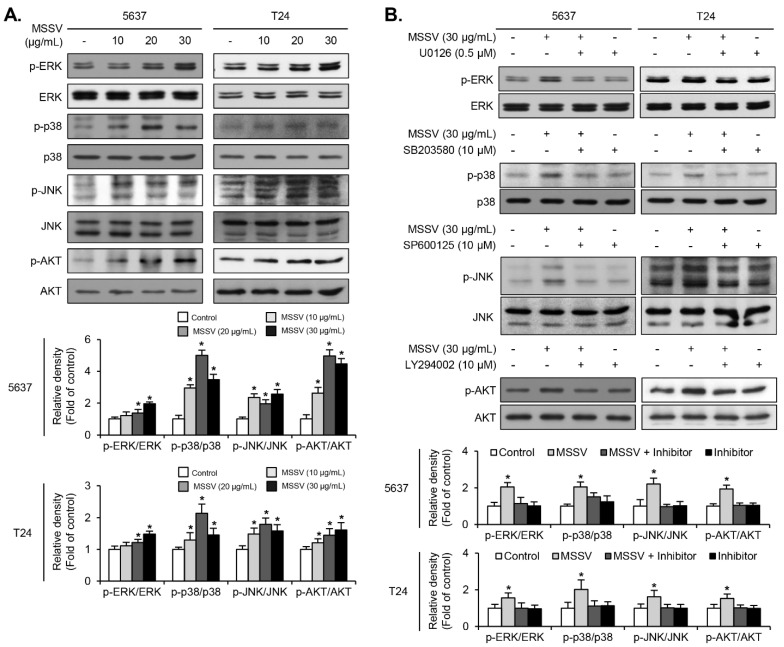
Signaling pathways mediating the anti-proliferative effect of MSSV in bladder cancer cells. (**A**) Both cells were treated with MSSV at the indicated concentrations for 24 h. The cell lysates were subjected to immunoblot analysis with phosphorylated and total forms of MAPKs (ERK1/2, JNK1/2, and p38) and AKT; Results were expressed as fold changes in comparison with the control cell lysates. (**B**) Both cells were pretreated with biochemical inhibitors of U0126 (0.5 μM), SB203580 (10 μM), SP600125 (10 μM), and LY 294002 (10 μM) for 40 min. MSSV was then added for another 24 h, followed by immunoblot analysis. The ratio of phosphorylated to non-phosphorylated form was measured and expressed as fold changes in comparison with the ratio associated with MSSV treatment. For the bar graphs, values are presented as the mean ± SD of three independent experiments; * *p* < 0.05, compared with the control group. Uncropped Western Blot Images in Figures S5 and S6.

To investigate whether the signaling pathway is involved in MSSV-mediated anti-proliferative effect of bladder cancer cells, we assessed the phosphorylation level of MAPKs (ERK1/2, p38MAPK, and JNK) and AKT in MSSV-treated cells. /The phosphorylation level of ERK1/2, p38MAPK, and JNK was upregulated in both bladder cancer cells treated with MSSV (Figure 3A). The level of AKT phosphorylation was also increased in both the MSSV-treated bladder cancer cells (Figure 3A). Additionally, the increased phosphorylation level of ERK1/2, p38MAPK, JNK, and AKT was suppressed by adding their specific inhibitors, U0126, SB203580, SP600126, and LY294002, respectively (Figure 3B). These results demonstrated that MAPKs and AKT signaling pathways played a role in the anti-proliferative effect of MSSV-treated bladder cancer cells.

### 3.6. MSSV Inhibits Wound Healing Migration and Invasion Abilities of Bladder Cancer Cells via Decreased MMP-9 Expression by Suppressing Transcription Factors

**Figure 4:**
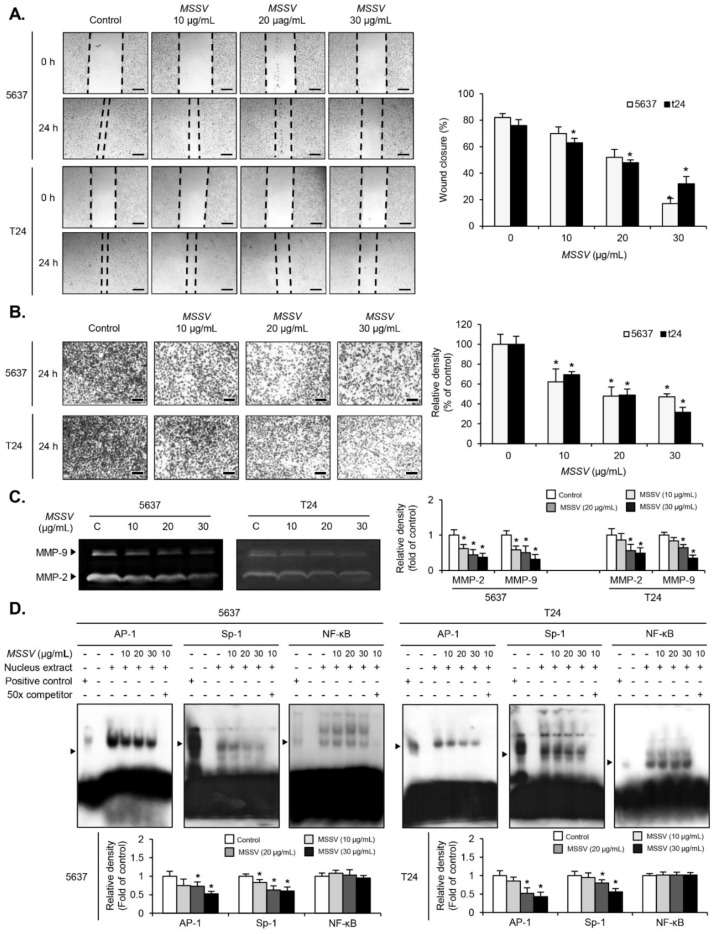
MSSV represses the migration and invasion of bladder cancer cells by suppressing transcription factor-mediated MMP-9 activity. Both 5637 and T24 cells were pretreated with mitomycin C, followed by incubation with MSSV for 24 h. (**A**) Cellular images of migratory recovery rate of 5637 and T24 cells photographed under an inverted microscope (scale bars = 200 µm). Bar graphs represent the relative fold changes in migration distances as compared with the control. (**B**) Treatment with MSSV inhibited the invasion ability of both bladder cancer cells (scale bars = 200 µm). Both cells were added onto the upper chamber and incubated with the indicated concentrations of MSSV for 24 h. Cells invading the lower surface of the membrane were detected by crystal violet staining. In the bar graphs, the amount of invading cells was assessed as the fold change compared with the control. (**C**) Zymographic assay was performed to determine MMP-9 expression in cells isolated from the cultured medium. Proteolytic activity of each MMP-9 was detected as a fold change compared with the control. (**D**) Nuclear extracts were subjected to EMSA assay to test the binding activities of activator protein 1 (AP-1), specificity protein 1 (Sp-1), and nuclear factor kappa-light-chain-enhancer of activated B cells (NF-κB) using radiolabeled oligonucleotide probes. Unlabeled AP-1, Sp-1, and NF-κB oligonucleotides were used as competitors. For the bar graphs, values are expressed as the mean ± SD of three independent experiments; * *p* < 0.05, compared with the control group.

We conducted the wound healing migration and invasion assays to evaluate the metastatic potential of MSSV in bladder cancer cells. In the wound healing assay, MSSV reduced the migration ability of both 5637 and T24 cells in a dose-dependent manner (Figure 4A). The wound closure rates of both bladder cancer cells treated with MSSV were lower than those of the untreated cells (Figure 4A). Additionally, transwell invasion chamber assay revealed that the number of penetrating MSSV-treated cells was significantly lower than that of the untreated cells (Figure 4B). Both wound healing migration assay and transwell chamber assay demonstrated that MSSV could inhibit the migration and invasion of bladder cancer cells. Given the inhibitory effect of MSSV on the migratory and invasive abilities of bladder cancer cells, we examined MMP-9 expression in the presence of MSSV. MMP-9 secretion in the conditioned medium was investigated by gelatin zymography in MSSV-treated bladder cancer cells. In comparison with the untreated cells, MSSV-treated bladder cancer cells had a lower level of MMP-9 secretion (Figure 4C). Similar patterns were observed for MMP-2 secretion (Figure 4C). Previous studies have demonstrated that the three main transcription factors, such as NF-κB, AP-1, and Sp-1, were involved in MMP-9 expression (3, 7). To further investigate the regulatory mechanism of MMP-9 in MSSV-treated bladder cancer cells, we employed EMSA assay. In both 5637 and T24 cells, MSSV treatment caused a significant reduction in the binding activity of AP-1 and Sp-1 but not NF-κB (Figure 4D). Thus, our data suggest that MSSV could impair the migration and invasion abilities of bladder cancer cells by suppressing transcription factor-mediated expression of MMP-9.

### 3.7. MSSV Induces Apoptosis by Regulating Apoptosis-Related Proteins in Bladder Cancer Cells

**Figure 5:**
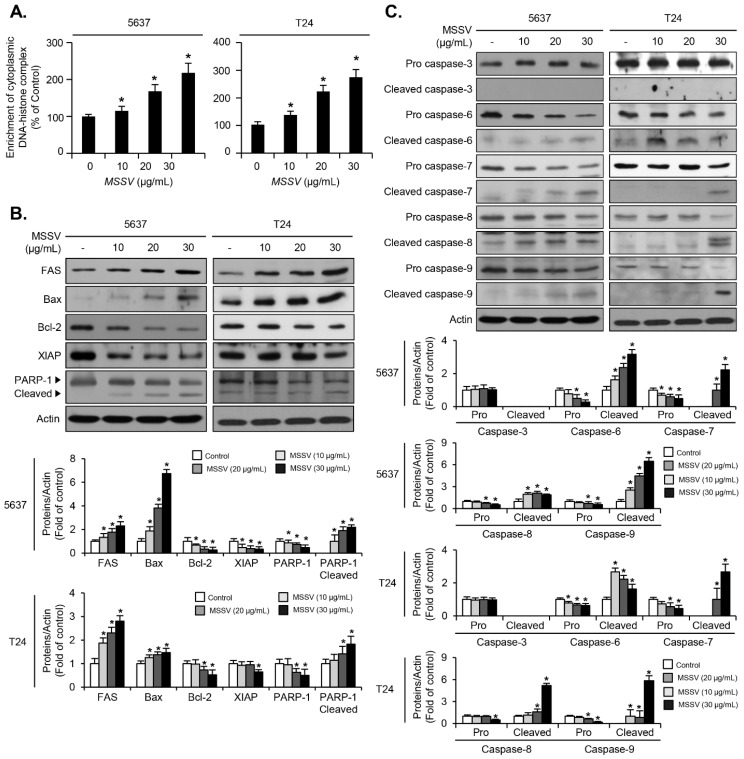
Effect of MSSV on apoptosis signaling in both bladder cancer cells. Both cells were exposed to MSSV at the indicated concentrations for 24 h. (**A**) Detection of apoptosis via measurement of cytoplasmic DNA-histone complex. After the treatment of cells with MSSV, supernatants containing cytoplasmic histone-associated DNA fragments were determined using ELISA assay. (**B**) Whole-cell lysates were subjected to immunoblot analysis with anti-FAS, anti-Bcl-2, anti-Bax, anti-XIAP, anti-PARP-1, and anti-Actin. (**C**) Expression levels of pro and cleaved forms of cascase-3, cascase-6, cascase-7, cascase-8, and cascase-9 were analyzed via immunoblotting. Actin was used as the internal control. Bar graphs show relative fold changes in the protein levels at different concentrations of MSSV compared with the control. For the bar graphs, values are presented as the mean ± SD of three independent experiments; * *p* < 0.05, compared with the control group. Uncropped Western Blot Images in Figures S7 and S8.

To investigate whether MSSV induces apoptosis in MSSV-treated bladder cancer cells, we performed apoptosis assay (determination of cytoplasmic histone-associated DNA fragments) after treating the cells with MSSV (0, 10, 20, or 30 μg/mL) for 24 h. Treatment with MSSV increased the cytoplasmic DNA–histone complex in both 5637 and T24 cells as opposed to that in the untreated cells (Figure 5A). To further analyze the mechanism underlying MSSV-induced apoptosis, we examined the essential regulators of apoptotic signaling in MSSV-treated bladder cancer cells. The present result showed that MSSV treatment induced FAS expression in both 5637 and T24 cells (Figure 5B). The expression of XIAP decreased in both of the MSSV-treated bladder cancer cells (Figure 5B). Cleavage of PARP-1 was observed in both MSSV-treated cells (Figure 5B). Additionally, it was found that upregulation of Bax expression occurred in both MSSV-treated 5637 and T24 cells, which led to reduction in Bcl-2 expression as opposed to the untreated cells (Figure 5B). Treatment with MSSV resulted in the activation of apoptosis signaling initiators, such as caspase-8 and caspase-9 in both cells (Figure 5C). Subsequently, the downstream effector caspases, including caspase-6 and caspase-7, were activated by MSSV treatment in both bladder cancer 5637 and T24 cells (Figure 5C). These results provide evidence for apoptosis induction in MSSV-treated bladder cancer cells.

### 3.8. Anti-Tumor Efficacy of MSSV in Human Bladder Tumor Xenograft Growth

**Figure 6:**
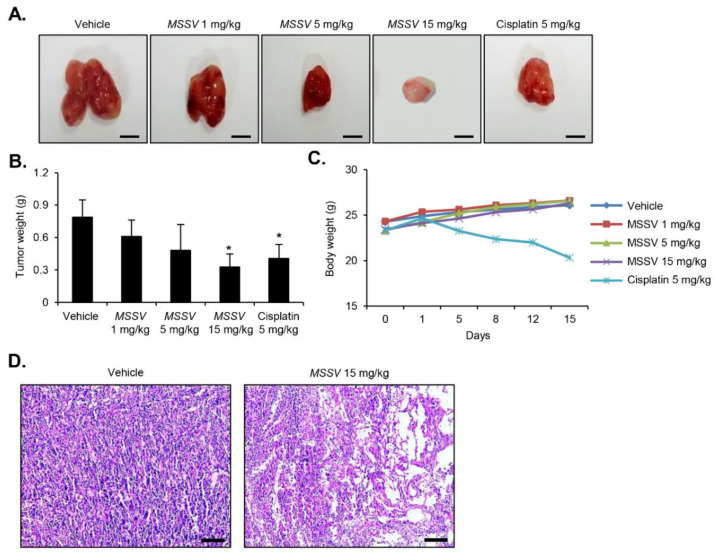
Antitumor efficacy of MSSV in bladder cancer 5637 xenograft mice models. (**A**) MSSV administration via oral gavage twice a day caused the inhibition of tumor growth in bladder cancer-xenografted mouse model (scale bars = 50 mm). (**B**,**C**) Tumor weight and body weight are represented in the bar graph. All data are represented as the means ± SE from three independent experiments. * *p* < 0.05, compared with control. (**D**) Tumor tissues were subjected to H&E staining for the detection of tumors (scale bars = 50 µm).

Based on the findings of MSSV-induced anti-tumor effect on bladder cancer cells *in vitro*, we used xenografted mice model implanted with 5637 cells to further investigate the anti-tumor efficacy of MSSV in vivo. Tumor weight was inhibited by 42% in mice that were orally administered with MSSV at 15 mg/kg as opposed to the tumor weight in control mice (Figure 6A,B). Moreover, there was no significant change in body weight for 16 days between MSSV-treated mice groups and control mice group (Figure 6C). However, decreased body weight and mice mortality were observed in cisplatin-treated groups, indicating that cisplatin was considerably toxic to mice (Figure 6C). The apoptotic tumor cells induced by MSSV were examined using H&E staining. The apoptosis of tumor cells was more evident in MSSV-treated mice models than in control mice (Figure 6D).

### 3.9. MSSV Inhibited VEGF-Induced Angiogenic Responses Both In Vitro and In Vivo

**Figure 7:**
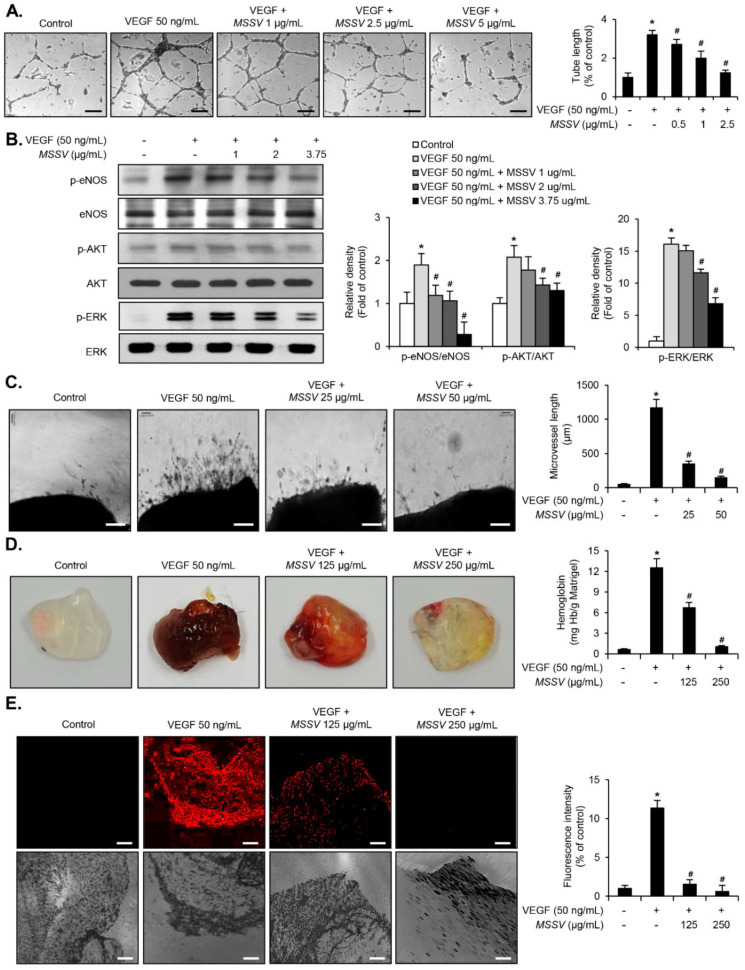
Effect of MSSV in VEGF-induced angiogenic responses in vitro, ex vivo, and in vivo. (**A**) HUVECs were treated with VEGF (50 ng/mL) for 1 h, followed by the indicated concentrations of MSSV for 24 h. The HUVEC tube formation assay was accomplished using Matrigel (scale bars = 200 µm). (**B**) Immunoblot analysis for antibodies specific for phospho-ERK1/2, ERK1/2, phospho-AKT, AKT, phospho-eNOS, and eNOS. (**C**) Blood neo-vessel sprouting in the mouse aorta after MSSV treatment in the presence of VEGF (50 ng/mL) was evaluated using aortic ring assay (scale bars = 200 µm). Bar graphs show the relative fold changes in the number of emerging neo-vessel associated with MSSV + VEGF treatment in comparison with that associated with VEGF treatment alone. (**D**) Blood vessel formation was performed using a Matrigel plug in an in vivo experiment. Blood vessel formation was assessed by measuring hemoglobin content in Matrigel. Bar graphs show the relative fold changes in the hemoglobin content associated with MSSV + VEGF treatment in comparison with that associated with VEGF treatment alone. (**E**) Analysis of vessel formation in Matrigel plug was performed by CD31 immunostaining (scale bars = 100 µm). Bar graphs show the relative fold changes in the density of CD31-positive vessels during MSSV + VEGF treatment as compared with that during VEGF treatment alone. All data are represented as the mean ± SE from three independent experiments. * *p* < 0.05 compared with control and ^#^ *p* < 0.05 compared with VEGF treatment. Uncropped Western Blot Images in Figure S9.

Next, our study focused on the effect of MSSV in VEGF-induced angiogenic responses. We found that MSSV inhibited proliferation, migration, invasion, and tube formation of VEGF-induced HUVECs (Figure 7A, Figure S1). Additionally, the treatment of HUVECs with MSSV led to a rapid reduction in the phosphorylation of eNOS, AKT, and ERK1/2 in VEGF-treated HUVECs (Figure 7B). To further investigate the anti-angiogenic effect of MSSV, we used an aortic ring ex vivo assay. As shown in Figure 7C, VEGF induced vessel sprouting from the aortic ring. This VEGF-induced microvessel emerging from the aortic ring was suppressed by MSSV treatment (Figure 7C). Furthermore, using a Matrigel plug in vivo assay, we confirmed the inhibitory effect of MSSV on angiogenesis. VEGF-supplemented Matrigel plugs exhibited a dark red color owing to the formation of blood vessels (Figure 7D), whereas the Matrigel plugs containing both MSSV and VEGF appeared light red in color, thereby demonstrating a reduction in blood vessel formation (Figure 7D). The extent of neovascularization in Matrigel plugs was determined via hemoglobin counting. MSSV treatment impeded the increase in hemoglobin content in Matrigel plugs in response to VEGF (Figure 7D). Microvessel density was confirmed by immunostaining with CD31 antibody (Figure 7E). These results suggest that MSSV suppresses the angiogenic responses induced by VEGF.

### 3.10. Acute Oral Toxicity of MSSV

The acute toxicity of MSSV (2000 mg/kg) was assessed by administering a single oral dose of MSSV over 14 days. There was no obvious difference in body weight, food intake, and water consumption between the control and MSSV-treated groups (Table S2). No deaths or adverse signs of toxicity were observed (Table S2). We subsequently evaluated the level of biochemical markers, AST and ALT, in serum. The levels of AST and ALT in both the male and female mice belonging to MSSV-treated groups were slightly lower than those in the control group (Figure S2A,B). H&E staining of liver tissues did not show any signs of inflammation and necrosis (Figure S2C,D). Taken together, statistically significant changes in toxic signs and biochemical parameters were not seen between the control and MSSV-treated animals, which may indicate that the hepatic function had not been impaired in both mice groups.

## 4. Discussion

In this study, we focused on MSSV as a strong anti-tumor agent. As revealed by cell counting and cell viability assay, MSSV treatment inhibited the proliferation of both T-24 and 5637 bladder cancer cell lines. Previous studies have reported that sansalvamide analogue (sansalvamide 12) suppressed pancreatic cell growth by arresting the G1-phase cell cycle [16]. Hence, we examined whether MSSV affects cell cycle regulation in bladder cancer cell lines. As expected, MSSV induced G1-phase cell cycle arrest in both T-27 and 5637 cell lines, which is associated with reduced expression of cyclin D1/CDK4 and cyclin E/CDK2 that are the two main protein complexes responsible for G1- to S-phase progression [4,5,7]. Investigation of both the MSSV-treated bladder cancer cell lines indicated an increase in p21WAF1 and p27KIP1 levels without an alteration in p53 level. These results demonstrated that MSSV suppresses the proliferation of bladder cancer cells via G1-phase cell cycle arrest by increasing p21WAF1 and p27KIP1 levels.

Apoptosis is known to regulate the response of cell death program [17,18]. Apoptosis is mainly divided into intrinsic pathway (Bcl-2 family/caspase-9/XIAP/caspase-3, or capsase-7/PARP-1 cascade) and extrinsic pathway (FAS/caspase-8XIAP//caspase-3 or capsase-7 cascade) [17,18]. Induction of tumor cell apoptosis is beneficial for the development of anti-cancer agents. The results of cell viability assay and immunoblot analysis as well as histone-complexed DNA fragments detection data revealed that MSSV treatment decreased the expression of Bcl-2 and XIAP in bladder cancer cells. Treatment of cells with MSSV caused an increase in Bax and Fas. Moreover, MSSV induced the activation of both initiator caspases (caspase-8 and -9) and effector caspases (caspase-6 and -7) as well as the proteolytic degradation of PARP. However, it is noteworthy that caspase-3 activation remained unaffected by MSSV treatment. The present data demonstrate the involvement of both intrinsic and extrinsic caspase pathways of apoptosis, which were associated with PARP-1 cleavage, Bax, and Fas induction, and Bcl-2 and XIAP suppression in a MSSV-treated bladder cancer cells.

Cumulating studies have demonstrated the involvement of MAPK and AKT signaling in cellular survival signaling [4,5,6]. In contrast, previous studies suggested that the inhibition of cell growth is mediated through the MAPK and AKT signaling pathway [4,7,19,20]. The results from the present study revealed that MSSV treatment induced the activation of AKT and MAPKs, such as ERK1/2, JNK, and p38MAPK. These results may highlight the critical view that AKT and MAPK signaling pathways are responsible for MSSV-induced inhibition of cell growth signal that counteracts the induction of G1-phase cell cycle arrest and the strong apoptotic effect. These data might suggest that AKT and MAPKs are crucial mediators of cell growth inhibition, G1-phase cell cycle arrest, and cell death signaling in MSSV-treated cells. However, further studies are warranted to understand the detailed mechanism underlying the interaction between the signaling pathway (AKT and MAPKs) and inhibitory cell growth (G1-phase cell cycle arrest and apoptosis) in bladder cancer cells treated with MSSV.

Both migration and invasion are believed to mediate progression and development of tumor cells [3,4,7]. MSSV treatment reduced the migration and invasion of bladder cancer cells. Matrix metalloproteinases (MMPs) are one of the main factors that cause the degradation of the surrounding extracellular matrix, which in turn activates the metastatic potential of cancer cells, including migration and invasion, through the basement membrane [3,4]. MMP-9 has been considered as a well-known proteolytic enzyme that plays a critical role in inducing migration and invasion of bladder cancer cells [3,4,7]. Additionally, transcription factors, including NF-κB, AP-1, and Sp-1, have been closely linked with MMP-9 expression in tumor cells [3,7]. The present results provide evidence that MSSV could impair MMP-9 expression by decreasing the binding activities of AP-1 and Sp-1 motifs, and thereby weakening the migration and invasion capabilities of bladder cancer cells.

Tumor growth warrants the formation of new vessels through angiogenesis [8,21]. VEGF is one of the most important angiogenic factors involved in the process of angiogenesis [22]. VEGF-induced tube formation, proliferation, migration, and invasion of endothelial cells are considered as pivotal mechanisms of angiogenesis [22,23,24,25]. eNOS, ERK1/2, and AKT signaling pathways are key events to regulate the endothelial cells in response to VEGF during angiogenesis [23,24,25]. Because angiogenesis is an important phenomenon for tumor growth, it is considered a suitable target in anti-tumor drug development strategies; we hypothesized that MSSV also demonstrates anti-angiogenic effects via suppression of VEGF-induced angiogenic responses. Here, we found that proliferation, migration, invasion, and tube formation are inhibited in VEGF-treated HUVECs. MSSV blocked the phosphorylation of eNOS, ERK1/2, and AKT induced by VEGF in HUVECs. Moreover, we observed the strong blockage of VEGF-induced neovessel formation by performing ex vivo aortic ring and in vivo Matrigel plug assays, which suggested that MSSV is a potent anti-tumor-associated angiogenic molecule.

Tumor-associated angiogenesis contributes to the growth, development, and establishment of solid tumors [8,21,22]. To further understand the anti-tumor effects of MSSV, we developed a xenograft mice model implanted with human bladder cancer 5637 cells. Oral administration of MSSV significantly suppressed the bladder tumor growth without altering body weight. The anti-tumor efficacy of MSSV (5 mg/kg) was similar to that of cisplatin drug (5 mg/kg). Particularly, 50% of the xenografted mice treated with cisplatin (5 mg/kg) died within 2 weeks. However, the xenografted mice that received oral administration of MSSV (15 mg/kg) remained alive for 2 weeks. These results led us to investigate whether a single oral dose of MSSV (2000 mg/kg) administered to mice over 14 days could be toxic. The present results of acute oral toxicity test show no signs of weight change and adverse clinical symptoms as well as no cases of animal death for both male and female mice. We subsequently investigated the biochemical levels of AST and ALT in serum, which are considered as suitable indicators of liver function; as expected, these biochemical tests along with histopathological analysis of liver tissue sections demonstrated that the oral administration of MSSV at 2000 mg/kg for 14 days did not cause any hepatic injury. Toxicity evaluation is one of the most essential steps for the development of an anti-tumor drug. Despite the identification of several cyclic depsipeptides including sansalvamide, *N*-methylsansalvamide, and their analogue [10,11,12,13,14], its safety level has never been addressed to date. As mentioned above, MSSV isolated from *Fusarium* spp. showed no evidence of acute toxic effects. These findings will help strategize the design and development of safer anti-cancer drugs having a strong pharmacologic activity. Further studies are required to clarify the cytotoxic effects on other tissues, such as the kidneys, testicles, heart, lungs, brain, and spleen.

## 5. Conclusions

In summary, our study is the first to demonstrate the therapeutic efficacy of MSSV as an anti-angiogenic and anti-tumor agent without any side effects by employing a primary disease model. Our preclinical data provide the evidence that MSSV is a potential therapeutic agent for bladder tumor. Further investigations are required to elucidate toxicity-related effects and pharmacodynamic characteristics of MSSV, which may be helpful in the clinical trial of patients with bladder cancer.

## Data Availability

Data is contained within the article or Supplementary Material.

## Supplementary Materials

The following are available online at https://www.mdpi.com/2072-6694/13/2/191/s1, Figure S1: MSSV inhibits VEGF-induced proliferation, migration, and invasion of HUVECs, Figure S2: Acute toxicity effects of MSSV on biochemical parameters in male and female mice, Figure S3: Uncropped Western Blot Images for Figure 2D, Figure S4: Uncropped Western Blot Images for Figure 2E, Figure S5: Uncropped Western Blot Images for Figure 3A, Figure S6: Uncropped Western Blot Images for Figure 3B, Figure S7: Uncropped Western Blot Images for Figure 5B, Figure S8: Uncropped Western Blot Images for Figure 5C, Figure S9: Uncropped Western Blot Images for Figure 7B, Table S1: 1H (400 MHz) and 13C (100 MHz) NMR data for N-methylsansalvamide in pyridine-d5, Table S2: Assessment of acute toxicity on oral consumption of NMSSA in male and female mice.

## References

[1] Metts M.C., Metts J.C., Milito S.J., Thomas C.R. (2000). Bladder Cancer: A Review of Diagnosis and Management. J. Natl. Med. Assoc..

[2] Shen Z., Shen T., Wientjes M.G., O’Donnell M.A., Au J.L. (2008). Intravesical Treatments of Bladder Cancer: Review. Pharm. Res..

[3] Black P.C., Dinney C.P.N. (2007). Bladder Cancer Angiogenesis and Metastasis—Translation from Murine Model to Clinical Trial. Cancer Metastasis Rev..

[4] Zachos I., Konstantinopoulos P.A., Tzortzis V., Gravas S., Karatzas A., Karamouzis M.V., Melekos M., Papavassiliou A.G. (2010). Systemic Therapy of Metastatic Bladder Cancer in the Molecular Era: Current Status and Future Promise. Expert Opin. Investig. Drugs.

[5] Yun S.J., Moon S., Kim W. (2013). Investigational Cell Cycle Inhibitors in Clinical Trials for Bladder Cancer. Expert Opin. Investig. Drugs.

[6] Zaravinos A., Lambrou G.I., Volanis D., Delakas D., Spandidos D.A. (2011). Spotlight on Differentially Expressed Genes in Urinary Bladder Cancer. PLoS ONE.

[7] Lee S., Cho S., Lee E., Kim S., Lee S., Lim J., Choi Y.H., Kim W., Moon S. (2013). Interleukin-20 Promotes Migration of Bladder Cancer Cells through Extracellular Signal-Regulated Kinase (ERK)-Mediated MMP-9 Protein Expression Leading to Nuclear Factor (NF-κB) Activation by Inducing the Up-Regulation of p21(WAF1) Protein Expression. J. Biol. Chem..

[8] Folkman J. (1995). Angiogenesis in Cancer, Vascular, Rheumatoid and Other Disease. Nat. Med..

[9] Seront E., Machiels J. (2015). Molecular Biology and Targeted Therapies for Urothelial Carcinoma. Cancer Treat. Rev..

[10] Lee H., Phat C., Choi S., Lee C. (2013). Synergistic Effect of a Novel Cyclic Pentadepsipeptide, neoN-Methylsansalvamide, and Paclitaxel on Human Multidrug Resistance Cancer Cell Lines. Anticancer Drugs.

[11] Sivanathan S., Scherkenbeck J. (2014). Cyclodepsipeptides: A Rich Source of Biologically Active Compounds for Drug Research. Molecules.

[12] Belofsky G.N., Jensen P.R., Fenical W. (1999). Sansalvamide: A New Cytotoxic Cyclic Depsipeptide Produced by a Marine Fungus of the Genus Fusarium. Tetrahedron Lett..

[13] Cueto M., Jensen P.R., Fenical W. (2000). N-Methylsansalvamide, a Cytotoxic Cyclic Depsipeptide from a Marine Fungus of the Genus Fusarium. Phytochemistry.

[14] Lee H., Lee C. (2012). Structural Analysis of a New Cytotoxic Demethylated Analogue of Neo-N-Methylsansalvamide with a Different Peptide Sequence Produced by Fusarium Solani Isolated from Potato. J. Agric. Food Chem..

[15] Park S.L., Chung T., Kim S., Hwang B., Kim J.M., Lee H.M., Cha H., Seo Y., Choe S.Y., Ha K. (2017). HSP70-1 is Required for Interleukin-5-Induced Angiogenic Responses through eNOS Pathway. Sci. Rep..

[16] Heiferman M.J., Salabat M.R., Ujiki M.B., Strouch M.J., Cheon E.C., Silverman R.B., Bentrem D.J. (2010). Sansalvamide Induces Pancreatic Cancer Growth Arrest through Changes in the Cell Cycle. Anticancer Res..

[17] Soldani C., Scovassi A.I. (2002). Poly(ADP-Ribose) Polymerase-1 Cleavage during Apoptosis: An Update. Apoptosis.

[18] de Almagro M.C., Vucic D. (2012). The Inhibitor of Apoptosis (IAP) Proteins are Critical Regulators of Signaling Pathways and Targets for Anti-Cancer Therapy. Exp. Oncol..

[19] Wang X., Martindale J.L., Holbrook N.J. (2000). Requirement for ERK Activation in Cisplatin-Induced Apoptosis. J. Biol. Chem..

[20] Vivanco I., Sawyers C.L. (2002). The Phosphatidylinositol 3-Kinase AKT Pathway in Human Cancer. Nat. Rev. Cancer.

[21] Hanahan D., Folkman J. (1996). Patterns and Emerging Mechanisms of the Angiogenic Switch during Tumorigenesis. Cell.

[22] Ferrara N. (2004). Vascular Endothelial Growth Factor: Basic Science and Clinical Progress. Endocr. Rev..

[23] Gerber H.P., McMurtrey A., Kowalski J., Yan M., Keyt B.A., Dixit V., Ferrara N. (1998). Vascular Endothelial Growth Factor Regulates Endothelial Cell Survival through the Phosphatidylinositol 3’-Kinase/Akt Signal Transduction Pathway. Requirement for Flk-1/KDR Activation. J. Biol. Chem..

[24] Pedram A., Razandi M., Levin E.R. (1998). Extracellular Signal-Regulated Protein Kinase/Jun Kinase Cross-Talk Underlies Vascular Endothelial Cell Growth Factor-Induced Endothelial Cell Proliferation. J. Biol. Chem..

[25] Dimmeler S., Fleming I., Fisslthaler B., Hermann C., Busse R., Zeiher A.M. (1999). Activation of Nitric Oxide Synthase in Endothelial Cells by Akt-Dependent Phosphorylation. Nature.

