# Supplementary material for: A Novel Cyclic Pentadepsipeptide, N-Methylsansalvamide, Suppresses Angiogenic Responses and Exhibits Antitumor Efficacy against Bladder Cancer
Source: Cancers (Basel). 2021 Jan 7;13(2):191. doi: 10.3390/cancers13020191 (PMC7827157; doi:10.3390/cancers13020191)
Supplement: Supplementary file 1 [file cancers-13-00191-s001.pdf]

# Supplementary Materials: A Novel Cyclic Pentadepsipeptide, *N*-methylsansalvamide, Suppresses Angiogenic Responses and Exhibits Antitumor Efficacy against Bladder Cancer

**Jun-Hui Song, Sung Lyea Park, Juhee Park, Byungdoo Hwang, Wun-Jae Kim, Chan Lee and Sung-Kwon Moon**

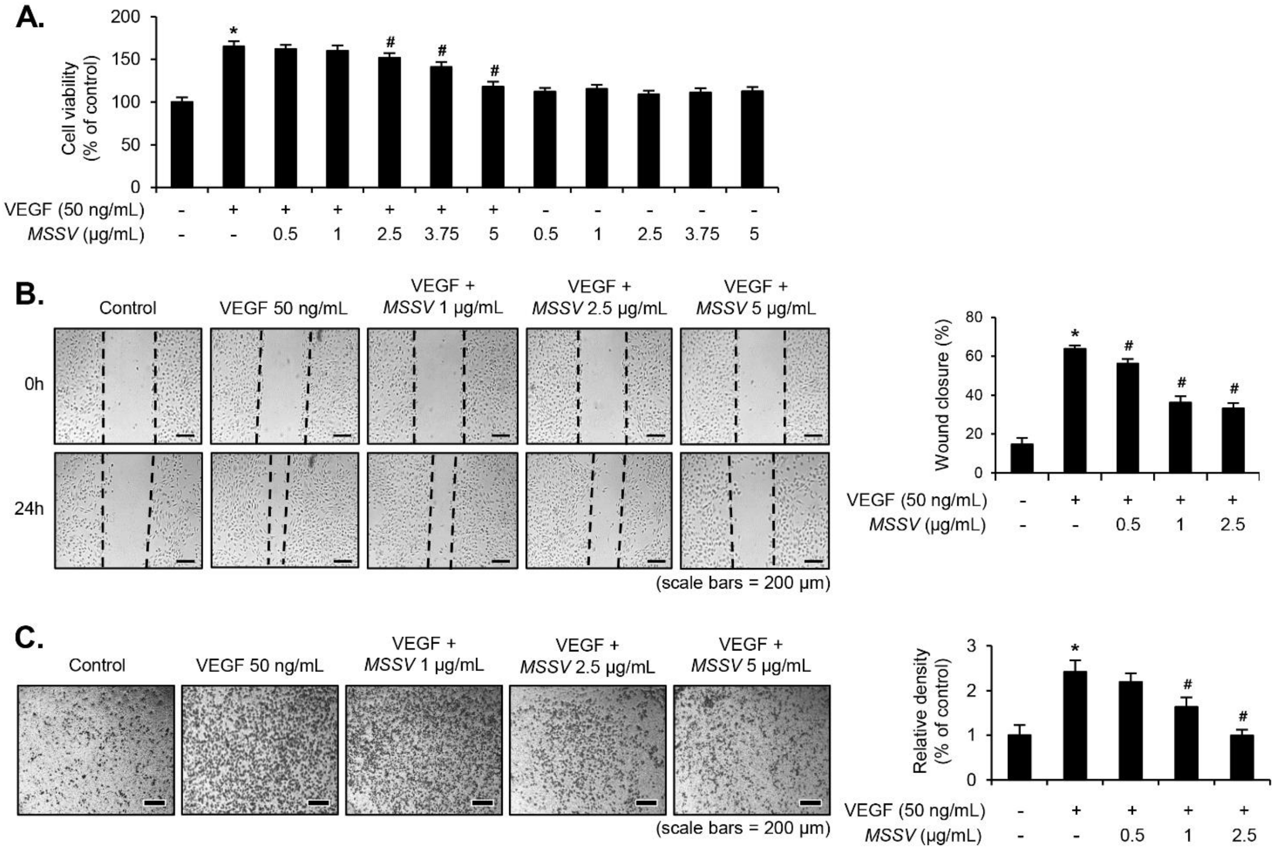


**Figure S1.** MSSV inhibits VEGF-induced proliferation, migration, and invasion of HUVECs. HUVECs were treated with VEGF (50 ng/mL) for 1 h, followed by the indicated concentrations of MSSV for 24 h. (**A**) MTT assay was performed after treatment with MSSV in the presence or absence of VEGF (50 ng/mL). (**B**,**C**) The migration and invasion abilities of HUVECs were analyzed by wound healing migration and Boyden chamber invasion assays. All data are reported as the means ± SE from three independent experiments. *$p < 0.05$ compared with control and #$p < 0.05$ compared with VEGF treatment.

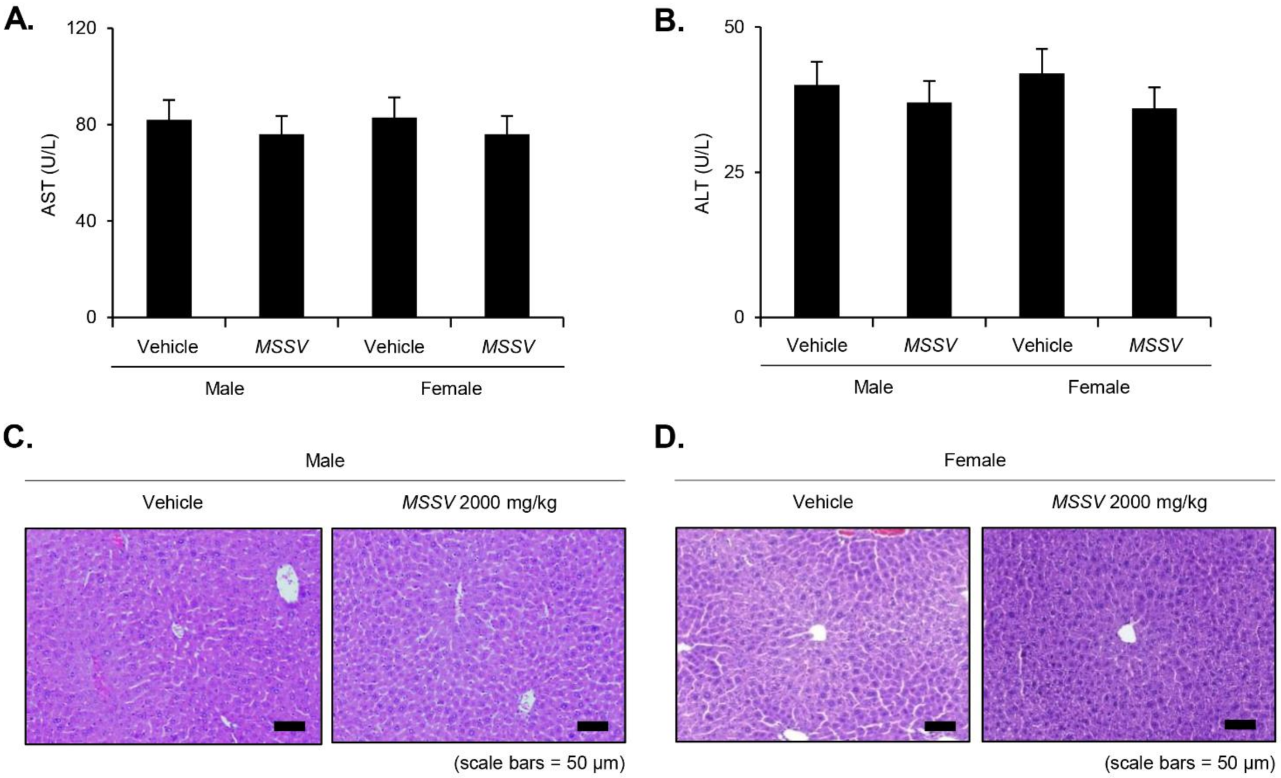


**Figure S2.** Acute toxicity effects of MSSV on biochemical parameters in male and female mice. After the oral administration of MSSV (2000 mg/kg), the levels of AST (**A**) and ALT (**B**) were measured. (**C,D**) H&E staining of the liver tissues obtained from MSSV-treated mouse. All data are represented as the means ± SE from three independent experiments. *$p < 0.05$ compared with the control.

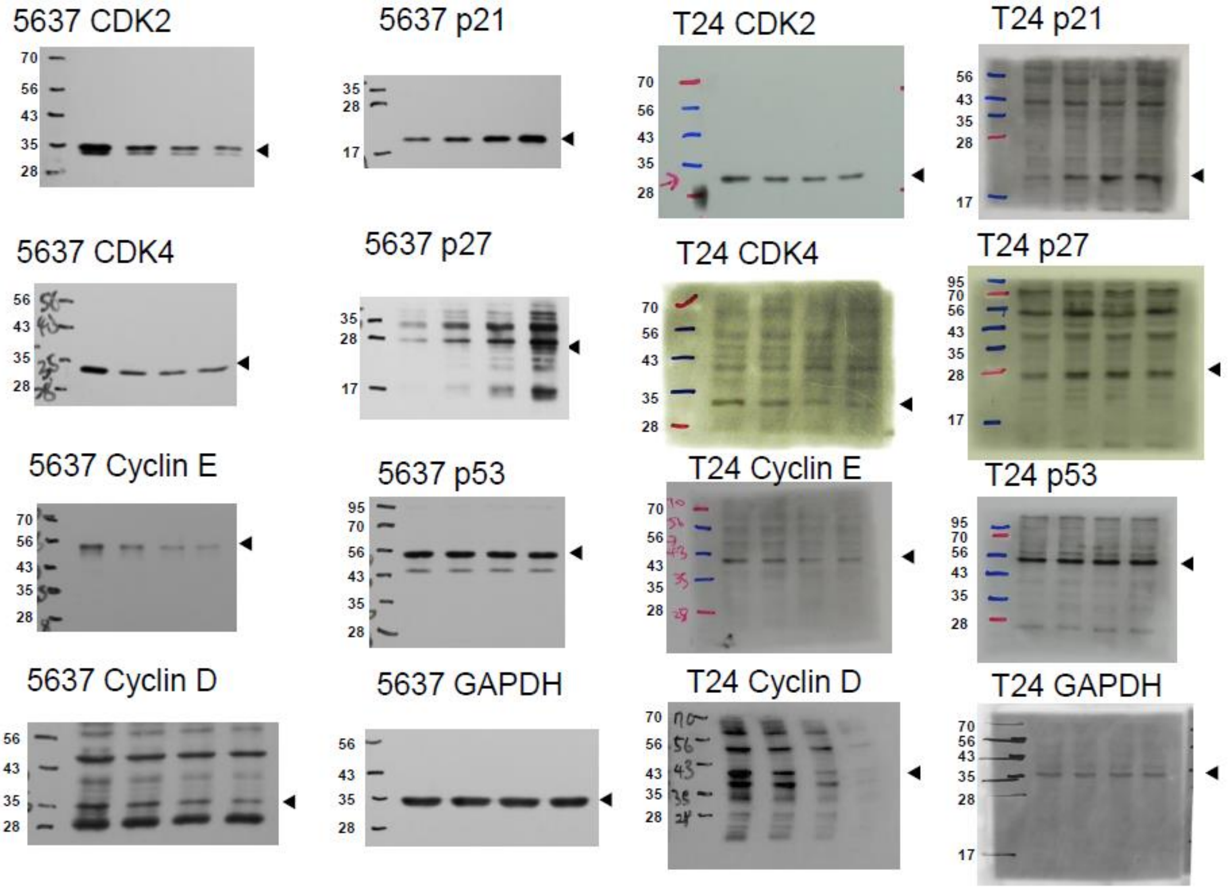

**Figure S3.** Uncropped Western Blot Images for Figure 2D.

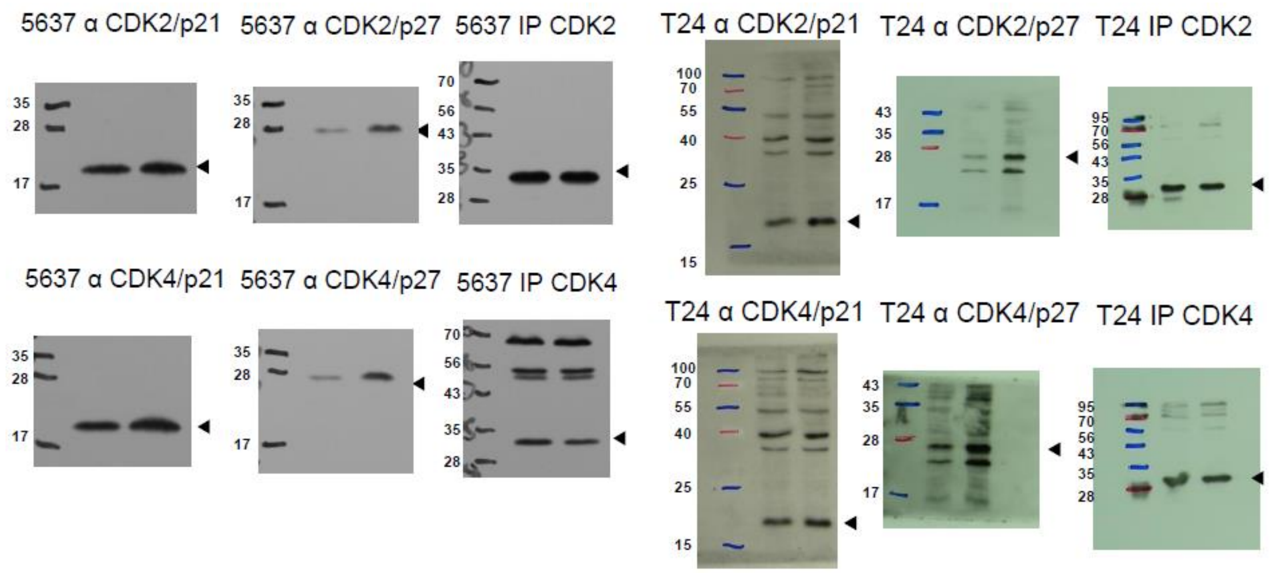


**Figure S4.** Uncropped Western Blot Images for Figure 2E.

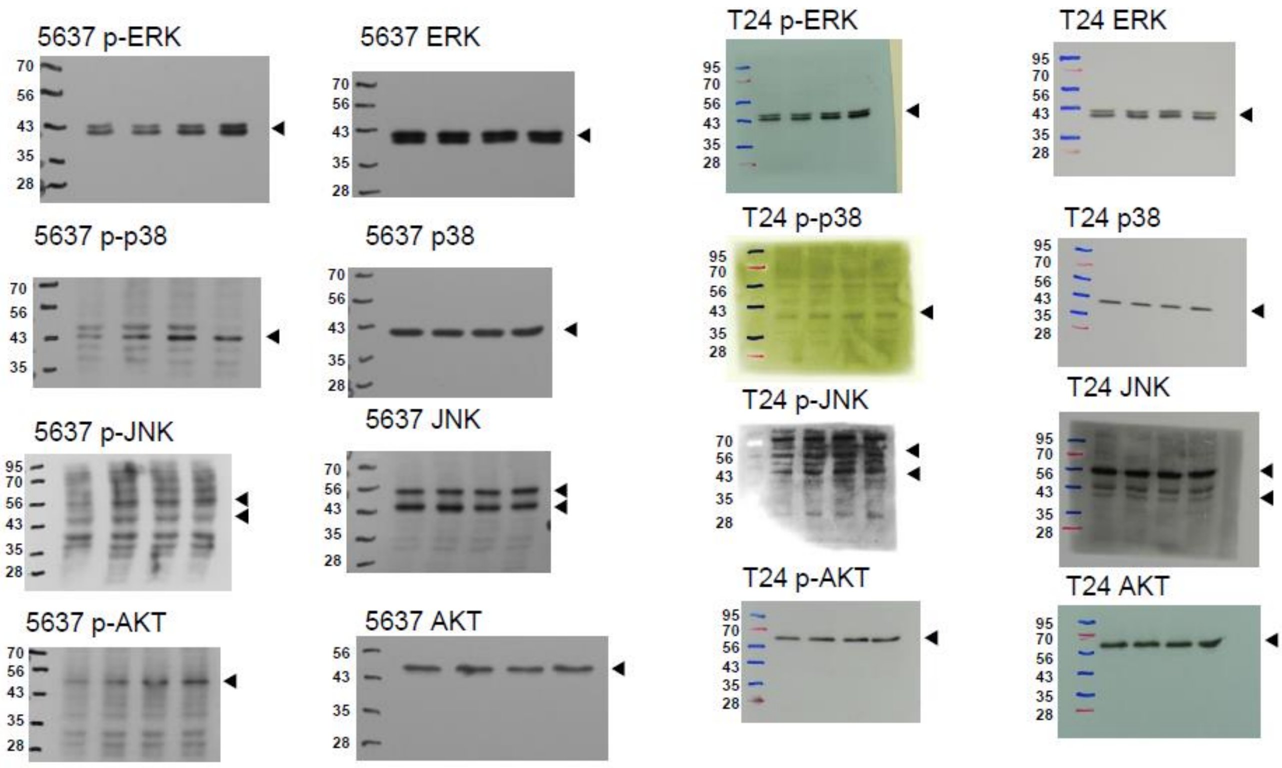


**Figure S5.** Uncropped Western Blot Images for Figure 3A.

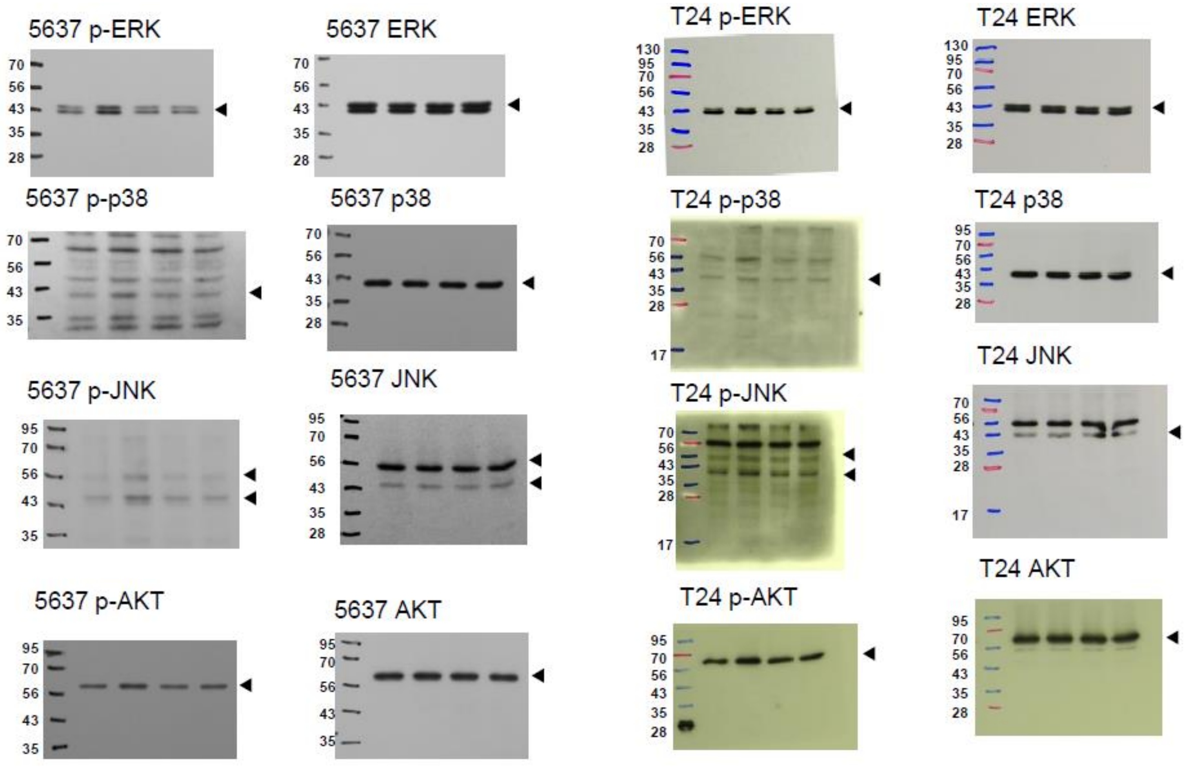


**Figure S6.** Uncropped Western Blot Images for Figure 3B.

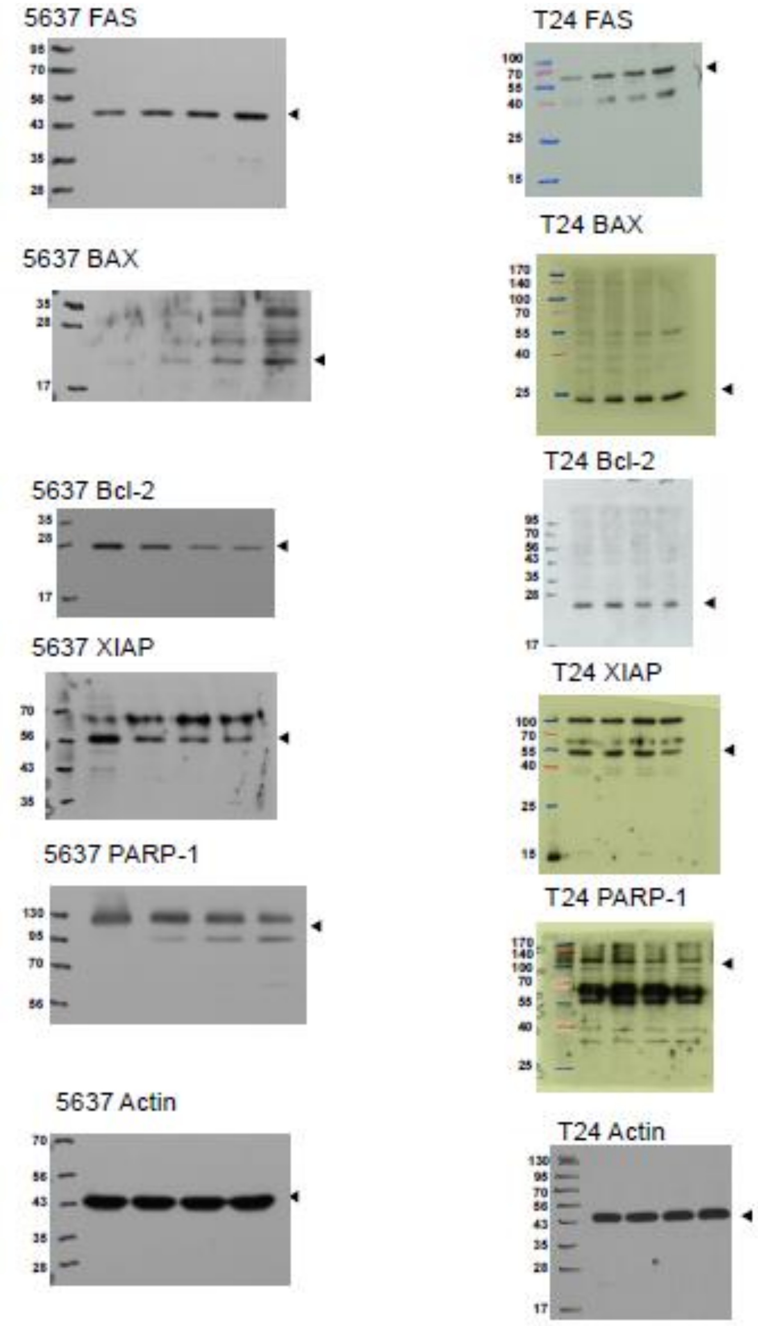


**Figure S7.** Uncropped Western Blot Images for Figure 5B.

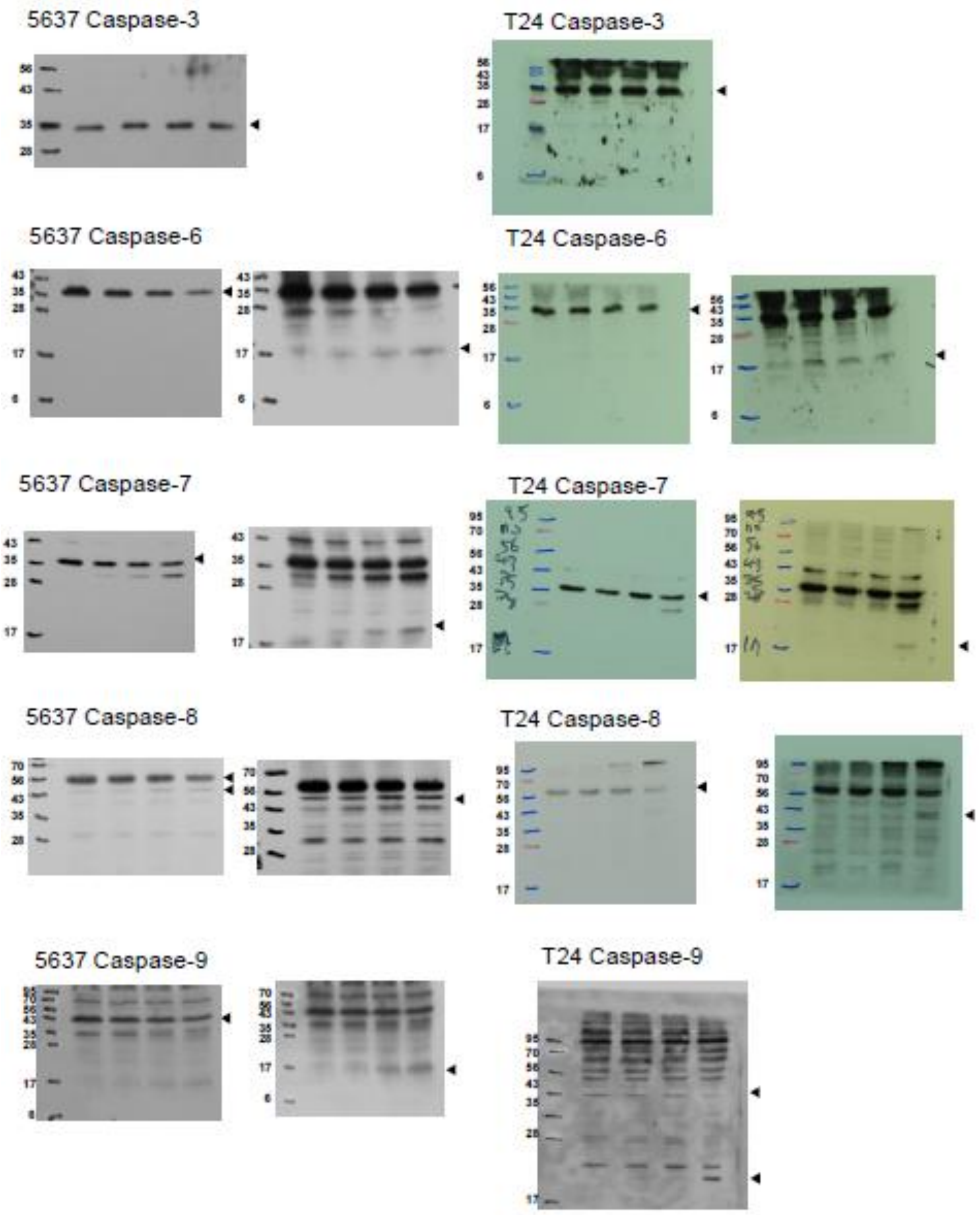


**Figure S8.** Uncropped Western Blot Images for Figure 5C.

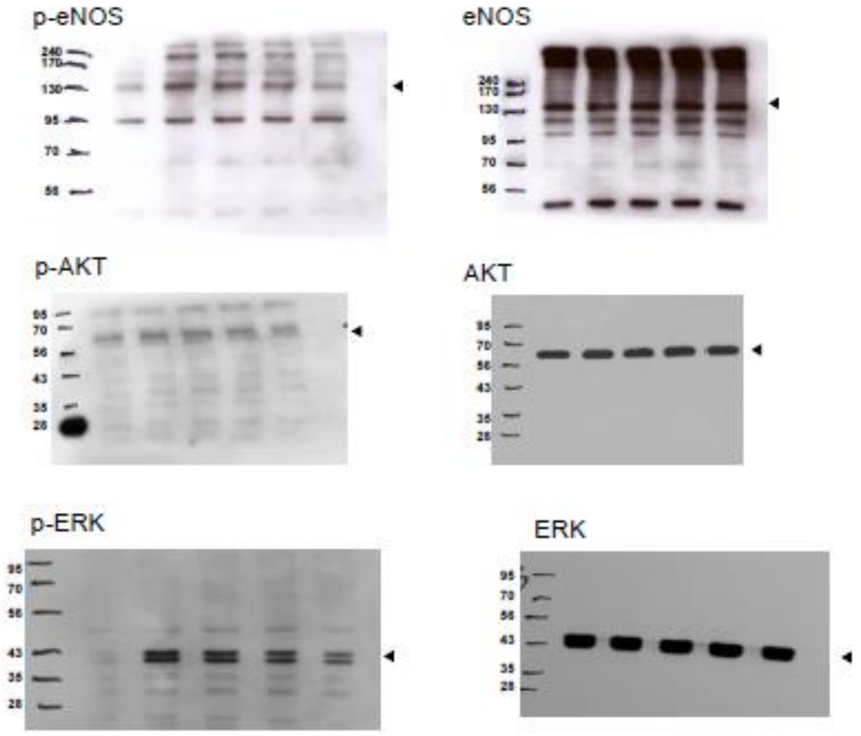


**Figure S9.** Uncropped Western Blot Images for Figure 7B.

**Table S1.** $^{1}$H (400 MHz) and $^{13}$C (100 MHz) NMR data for N-methylsansalvamide in pyridine-$d_5$.

| Position | | $\delta_C$, mult. | $\delta_H$, (*J* in Hz) |
|---|---|---|---|
| Valine | NH | | 7.76 (d. 8.9) |
| | *α* | 55.5, CH | 4.97 (dd. 8.9, 6.3) |
| | *β* | 31.1, CH | 2.31 (m) |
| | *γ* | 20.6, $CH_3$ | 1.01 (d, 6.8) |
| | *γ′* | 17.7, $CH_3$ | 1.11 (d, 6.6) |
| | CO | 173.2, C | |
| N-methyl Leu | N-$CH_3$ | 39.9 | 3.18 (s) |
| | *α* | 68.4, CH | 3.71 (dd. 9.0, 6.6) |
| | *β* | 39.7, $CH_2$ | 2.31[a], 2.06[a] |
| | *γ* | 26.1, CH | 1.60 (m) |
| | *δ* | 24.0, $CH_3$ | 0.83 (d, 6.3) |
| | *δ′* | 23.6, $CH_3$ | 0.92 (d, 6.6) |
| | CO | 172.2 | |
| Phenylalanine | NH | | 8.68 (d, 9.0) |
| | *α* | 56.5, CH | 5.10 (td. 9.1, 6.4) |
| | *β* | 39.7, $CH_2$ | 3.54 (dd, 13.8, 6.4), 3.39 (dd, 13.8, 9.2) |
| | C1 | 138.9, C | |
| | C2 | 129.4, CH | 7.30[a] |
| | C3 | 129.3, CH | 7.32[a] |
| | C4 | 127.4, CH | 7.28[a] |
| | CO | 172.9, C | |
| Leucine | NH | | 9.45 (d, 8.6) |
| | *α* | 52.6, CH | 4.87 (ddd, 10.9, 8.5, 4.6) |
| | *β* | 38.3, $CH_2$ | 2.22[a], 1.93[a] |
| | *γ* | 25.0, CH | 1.50[a] |
| | *δ* | 22.4, $CH_3$ | 0.94 (d, 6.3) |
| | *δ′* | 23.3, $CH_3$ | 0.88 (d, 6.4) |
| | CO | 172.0[a] | |
| 2-hydroxy-4-methyl pentanoic acid | C2 | 76.3, CH | 5.35 (dd, 9.6, 3.7) |
| | C3 | 41.5, $CH_2$ | 1.91[a] |
| | C4 | 25.6, CH | 2.05[a] |
| | C5 | 21.7[a], $CH_3$ | 0.94[a] |
| | C6 | 22.1[a], $CH_3$ | 0.92[a] |
| | CO | 171.4[a] | |

[a]Overlapped signals.

**Table S2.** Assessment of acute toxicity on oral consumption of *MSSV* in male and female mice.

| Group | Dose (mg/kg) | Volume (mL/kg) | Number of Animals | | Symptom | Autopsy |
|---|---|---|---|---|---|---|
| | | | Male | Female | | |
| Control | 0 | 4 | 5 | 5 | Normal | Normal |
| *MSSV* | 2000 | 4 | 5 | 5 | Normal | Normal |

**Publisher's Note:** MDPI stays neutral with regard to jurisdictional claims in published maps and institutional affiliations.

**Copyright:** © 2021 by the authors. Licensee MDPI, Basel, Switzerland. This article is an open access article distributed under the terms and conditions of the Creative Commons Attribution (CC BY) license (http://creativecommons.org/licenses/by/4.0/).
